# Real-Life Cause-Effect Relations Between Urinary IL-6 Levels and Specific and Nonspecific Symptoms in a Patient With Mild SLE Disease Activity

**DOI:** 10.3389/fimmu.2021.718838

**Published:** 2021-12-17

**Authors:** Christian Schubert, Lennart Seizer, Emil Chamson, Paul König, Norbert Sepp, Francisco M. Ocaña-Peinado, Mirjam Schnapka-Köpf, Dietmar Fuchs

**Affiliations:** ^1^ Department of Psychiatry, Psychotherapy, Psychosomatics and Medical Psychology, Medical University Innsbruck, Innsbruck, Austria; ^2^ Department of Translation Studies, Leopold-Franzens-University, Innsbruck, Austria; ^3^ Clinical Department of Internal Medicine, Medical University Innsbruck, Innsbruck, Austria; ^4^ Department of Dermatology, Ordensklinikum Linz, Elisabethinen, Linz, Austria; ^5^ Department of Statistics and Operations Research, University of Granada, Granada, Spain; ^6^ Central Institute of Medical and Chemical Laboratory Diagnostics, University Clinics, Innsbruck, Austria; ^7^ Division of Biological Chemistry, Biocenter, Medical University Innsbruck, Innsbruck, Austria

**Keywords:** lupus, interleukin-6, proteinuria, oral ulcer, facial rash, integrative single-case design, time-series analysis

## Abstract

**Background:**

Little is known about the real-time cause-effect relations between IL-6 concentrations and SLE symptoms.

**Methods:**

A 52-year-old woman with mild SLE activity collected her entire urine for the determination of IL-6/creatinine and protein/creatinine levels (ELISA, HPLC) for a period of 56 days in 12 h intervals (total: 112 measurements). Additionally, she answered questionnaires (VAS) on oral ulceration, facial rash, joint pain, fatigue and tiredness and measured her temperature orally twice a day. Time-series analyses consisted of ARIMA modeling and cross-correlational analyses (one lag = 12 h, significance level = *p* < 0.05).

**Results:**

Statistical analyses showed that increased urinary IL-6 concentrations preceded increased urinary protein levels by 36–48 h (lag3: r=+.225; *p*=.017) and that, in the opposite direction of effect, increased urinary protein preceded urinary IL-6 decreases by 12–24 h (lag1: r=–.322; *p*<.001). Moreover, urinary IL-6 increases co-occurred with increased oral ulceration (lag0: r=+.186; *p*=.049); after 48–60 h, however, IL-6 increases showed a strong tendency to precede oral ulceration decreases (lag4: r=–.170; *p*=.072). Increases in facial rash preceded decreases in urinary IL-6 after 84–96 h (lag7: r=–.215; *p*=.023). As to fatigue, increases in urinary IL-6 co-occurred with decreased fatigue (lag0: r=–.193; *p*=.042); after 84–96 h, however, IL-6 increases preceded fatigue increases (+lag7: *r*=+.189; *p*=.046). Finally, joint pain, tiredness and body temperature did not significantly correlate with urinary IL-6 concentrations in either direction of effect.

**Conclusions:**

The results of this evaluation point to real-life feedback mechanisms between immune activity and SLE symptoms. Comparison with a previous evaluation of this patient suggests a counterregulatory mechanism between Th1 activity and IL-6. These findings are preliminary and require replication to draw firm conclusions about the real-time relation between IL-6 and SLE disease activity.

## Introduction

The functional role of interleukin-6 (IL-6) in systemic lupus erythematosus (SLE) is uncertain and in need of clarification. IL-6 is a pleiotropic cytokine released into circulation in almost all situations of perturbation of the homeostasis of the organism (1). Moreover, IL-6 is a broad-spectrum cytokine that plays a role in various biological activities. It is involved not only in the activation of the immune system but also in regenerative processes, in the regulation of metabolism, in the maintenance of bone homeostasis and in many neural functions (2).

IL-6 is therefore a good candidate when looking for crucial pathogenic players involved in the protean clinical outcomes of human SLE (3). However, results on the connection between IL-6 and clinical manifestations of SLE have been inconsistent. Some investigations have revealed that elevated IL-6 levels reflect disease activity including American College of Rheumatology (ACR)-based symptoms such as fatigue, joint pain, proteinuria, fever, photosensitivity, rash and renal disorder (4), whereas others have failed to find any significant correlations (5).

An important aspect in the immunopathophysiology of SLE is an imbalance in T helper type 1 (Th1) and T helper type 2 (Th2) subsets as well as a dysregulation between T effectors (Th1, Th2, T helper type 17 [Th17]) and T regulatory (Treg) cells (6, 7). However, as with the literature on the association between IL-6 and SLE symptoms, previous reports on Th1/Th2 ratio in SLE have been inconsistent, with some studies describing a predominance of Th1 cytokines (8, 9), while others have reported a predominance of Th2 cytokines (10, 11). Therefore, a mutual contribution of Th1/Th2 ratio to SLE pathology has been hypothesized, with different cytokine patterns at different time points (12).

We propose that the divergent findings concerning IL-6 and SLE as well as Th1/Th2 ratio and SLE may be related to fundamental methodological problems associated with the negligence of the highly dynamic character of IL-6 (13) and of SLE symptoms (14). Conventional methodological approaches are designed to reveal whether variables are concurrently related or not and therefore focus on absolute values rather than on temporal relations between consecutive realizations of variables. Consequently, such research designs cannot properly deal with questions of the temporal delay of cause-effect relations between variables and the temporal pattern of such relations (15). Moreover, IL-6 and SLE symptoms may influence each other in both directions of effect (16), again something that cannot be targeted by conventional methodology (15).

Such cause-effect relations between cytokines and lupus symptoms have already been shown for soluble tumor necrosis factor receptor 55kD (sTNF-R55) – a Th1 cytokine associated with clinical and subclinical SLE disease activity (17) – in a previous study applying the integrative single-case design (18). The integrative single-case design is different from conventional methodology in that it uses time-series analysis and qualitative tools to investigate real-life cause-effect relationships between various biological, psychological and social variables under conditions of “life as it is lived” (15). The study mentioned above was conducted with a 52-year-old woman with infrequently occurring, minor SLE symptoms not requiring steroidal or immunosuppressive drug therapy. In order to preserve the patient’s normal routine as much as possible, proteinuria and cytokine levels were determined in 12 h urine samples to serially monitor these parameters non-invasively. Moreover, instead of having to see a physician every 12 hours, the patient used 100 mm visual analogue scales (VAS) to self-determine the presence of several specific (i.e. oral ulcers, facial rash and joint pain) and nonspecific SLE symptoms (i.e. fatigue, tiredness and body temperature). This procedure contributes to the high ecological validity of the study design.

In that study, we found that sTNF-R55 showed bidirectional cause-effect relations when cross-correlated with SLE symptoms (18). In particular, increased urinary sTNF-R55 concentrations preceded decreased urinary protein levels by 36–48 h, and, in the opposite direction of effect, increased urinary protein levels preceded increased urinary sTNF-R55 concentrations by 24–36 h. Furthermore, increases in urinary sTNF-R55 levels preceded increases in oral ulcers by 36–48 h, and increases in oral ulceration preceded decreases in urinary sTNF-R55 levels by 36–48 h. These cross-correlations in both directions of effect indicate feedback loops between sTNF-R55 and SLE symptoms. For example, elevated sTNF-R55 levels may have inhibited clearance of protein from circulation, while decreased protein clearance may have then resulted in decreased sTNF-R55 concentrations either per se or *via* an as yet unknown counterregulatory mechanism (18).

Similar bidirectional mechanisms might characterize the relationship between IL-6 and SLE symptoms. Both TNF-α and IL-6 are able to regulate the Th1/Th2/Th17/Treg balance, which plays a prominent role in autoimmune pathogenesis (6) *via* feedback loops, and therefore contribute to the maintenance of immunological homeostasis (19, 20).

The present article deals with a re-evaluation of the above-mentioned integrative single-case study and takes advantage of the opportunity to not only cross-correlate IL-6 and SLE symptoms (i.e. proteinuria, oral ulcers, facial rash, joint pain, fatigue, tiredness, body temperature) in the same patient but also to compare them with the findings on the relation between sTNF-R55 and SLE symptoms described above (18). Ultimately, this research strategy allows us to investigate the potentially diverse functional roles of sTNF-R55 and IL-6 in SLE.

## Patient and Methods

### Study Design

At study start, the patient was thoroughly examined psychologically as well as physically, the latter to ensure that she was in clinical remission (according to the Systemic Lupus Activity Measure [SLAM]). Then, during the following 56 days, the patient collected her entire urine in 12 h intervals (from approx. 8 a.m. to 8 p.m. and from approx. 8 p.m. to 8 a.m.; total: 112 time intervals) in two canisters per day (containing 0.5 g Na-Metabisulfite and 0.5 g Na-EDTA to prevent urine sedimentation and oxidation) and froze aliquoted urine samples at –20°C. She also filled out questionnaires twice a day at approx. 8 a.m. and 8 p.m. Each week, the patient brought the frozen urine samples to the laboratory where they were stored at –70°C. During each of these weekly visits, an in-depth psychological interview was conducted to identify the previous week’s incidents. In addition, a physical examination including a hemogram was performed to check general health and signs of SLE disease activity (SLAM). A more detailed description of the study design is given in (21).

### Patient Description and Disease History

The patient is a 52-year-old white post-menopausal woman and a non-smoker. Eight years prior to the study start in 1997, the diagnosis SLE was made by a senior internist (P.K.) and a senior dermatologist (N.S.) according to the following ACR criteria: kidney involvement (histological evaluation of chronic mesangial proliferative glomerulonephritis, WHO classification IIIa) with microscopic hematuria; arthralgia; urticarial vasculitis; oral ulcers; facial rash. Moreover, she showed decreased complement C4 (hypocomplementemia), leukopenia and enhanced antinuclear antibodies (ANA, 1:2560); analyses of antinuclear anti-double-stranded DNA antibodies (ds DNA) were negative.

Pharmacologic treatment lasted three years and consisted primarily of steroids (4–20mg) in combination with other non-steroidal anti-inflammatory medication (paracetamol). The patient did not tolerate antimalarials; moreover, she refused further immune suppressive therapy (e.g. azathioprine, mycophenolate, cyclophosphamide) although her disease fulfilled WHO classification IIIa for SLE. Nevertheless, her laboratory values improved (no proteinuria, no pathological urine sediment) during pharmacologic treatment. The patient attended psychotherapy for three years following diagnosis.

During regular check-ups between first diagnosis in 1989 and study start in 1997, the following minor clinical disease manifestations related to SLE had been identified: oral ulcers, urticarial vasculitis lesions at various body sites (e.g. facial rash), small joint pain, fatigue, tiredness and fever. These symptoms did not require steroidal or immunosuppressive drug therapy and were treated by the patient symptomatically (e.g. mouth rinsing with hexetidin solution). At study start, the patient presented with elevated ANA (1:160, ds DNA negative, SS-Ro-antibody positive) with the above-mentioned mild clinical symptoms, which did not require steroid treatment.

### Measurement of Small Joint Pain, Oral Ulceration, Facial Rash, Fatigue, Tiredness, and Body Temperature

In the morning and in the evening (i.e. in 12 h intervals), the patient used VAS/notes to indicate the following: small joint pain; mucosal and cutaneous manifestations such as oral ulcers and facial rash; fatigue; and tiredness. These measurements are part of the DIARI, a paper-and-pencil questionnaire that also includes drug/medication use and potential signs of a cold, flu, etc. (15). In addition, she measured her body temperature orally within 120 sec. using a commercially available mercury thermometer with a scale interval of 0.1°C (model no. 1711, Scheiber GmbH, Kreuzwertheim, Germany). The data were used to construct time series dealing with small joint pain, oral ulceration, facial rash, fatigue, tiredness and body temperature.

### Measurement of Urinary IL-6 and Creatinine Levels

Urine samples were stored at –70°C until analysis. We measured the 112 consecutive urinary IL-6 levels in one single run using ELISA as recommended by the manufacturer (Endogen, IBL, Hamburg). Urinary IL-6 concentrations were expressed in microgram per molar (μg/mol) creatinine. Urinary creatinine levels were measured applying High Pressure Liquid Chromatography (HPLC) (Model LC 550; Varian Associates, Palo Alto, CA) as previously described (22). We used a new aliquot for each of the three independent determinations.

### Measurement of Urinary Protein

The urinary protein level in each 12 h urine sample was measured in milligram per deciliter (mg %) using the benzethonium chloride method (23) at 505 nm with a Hitachi 911 analyzer (Roche). Values were expressed as miligram per micromolar (mg/μmol) creatinine (HPLC).

### Time-Series Analysis

A detailed description of the statistical analyses used in this study is given in (15). In short, cross-correlational analyses between IL-6 levels and the ACR criteria under study were performed at lag0 and at higher lags up to +/–7 using SPSS-Trends™ 26.0 (24). We controlled for spurious cross-correlations due to trends (e.g. circadian rhythm) and serial dependencies (e.g. autoregression) by cross-correlating residuals series after autoregressive integrated moving average (ARIMA) modeling of time series (25). In case the mean of a series needed to be stabilized, a deterministic trend was either removed from the series, or the series was differenced. In case the variance of a series needed to be stabilized, the series was transformed (e.g. log, square root). Transformation of time series was also used to improve model specification. Moreover, time series which did not need to be modeled and which were found to be not normally distributed were transformed before cross-correlating. Based on experience with our previous studies, binary time series were not modeled. Time series with missing values were linearly interpolated before further analysis. The level of statistical significance was set at *p* < 0.05.

## Results

**Figure 1:**
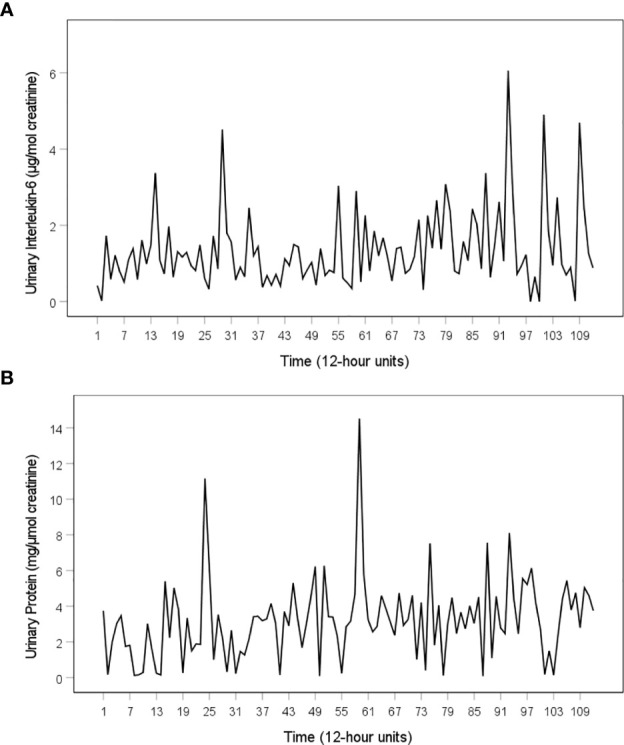
Time series of urinary IL-6 levels and urinary protein levels of the SLE patient under study. **(A)** Time series of urinary IL-6 (µg per mol creatinine), **(B)** Time series of urinary protein (μg per mol creatinine). Both time series cover a period of 56 days. During this time, the patient collected her full urine output in 12 h intervals, resulting in a total of 112 12 h measurements. The 112 12 h units consist of daytime intervals (from 8:00 a.m. to 8:00 p.m., uneven numbers) and nighttime intervals (from 8:00 p.m. to 8:00 a.m., even numbers).

Mean, standard deviation and range of all variables under study are shown in **Table 1**. All time series were complete (i.e. 112 measurements) except for the tiredness time series, which had one missing value (at 12 h unit 52). The patient indicated a mild facial rash on 26 of 112 12 h units (23%) (maximum intensity: 22%; maximum duration: 60 h). Moreover, she had mild oral ulcers (maximum intensity: 19.9%; maximum duration: 24h) on 6 of 112 12 h units (5%). Facial rash and oral ulcers were expressed in binary time series. **Figures 1A, B** show the time series of urinary IL-6 concentrations (μg/mol creatinine) and urinary protein concentrations (mg/μmol creatinine). In the middle of the study period (during 12 h units 45–54), the patient was diagnosed with acute paranasal sinusitis (21). Comparison of grouped time series data using Mann-Whitney U test revealed that only tiredness levels differed significantly during sinusitis compared to before and after sinusitis. The time series of tiredness remained heteroskedastic even after log transformation (data not shown). Neither the weekly clinical check-ups nor the 12 h notes taken by the patient revealed any signs of infection and/or SLE exacerbation during the study period.

**Table 1:** Descriptive statistics of urinary IL-6 concentrations and SLE-specific and SLE-nonspecific symptoms (N=112 consecutive measurements).

Parameter	Mean ± SD	Range
Urinary IL-6 (μg/mol creatinine)	1.36 ± 1.04	0.00 – 6.06
Urinary IL-6 (pg/ml)	0.01 ± 0.007	0.00 – 0.04
Urinary IL-6 (pg/h)	0.79 ± 0.67	0.00 – 4.20
Urinary protein (mg/μmol creatinine)	3.20 ± 2.25	0.08 – 14.5
Urinary protein (mg/dl)	3.48 ± 2.58	0.10 – 10.0
Urinary protein (mg/h)	1.76 ± 1.09	0.07 – 6.38
Oral ulceration (%)	0.59 ± 2.79	0.00 – 19.9
Facial rash (%)	2.67 ± 5.60	0.00 – 22.0
Body temperature (°C)	36.7 ± 0.25	36.1 – 37.4
Tiredness (%)	7.77 ± 5.86	0.00 – 38.0
Fatigue (%)	35.2 ± 17.2	6.00 – 77.0
Joint pain (%)	20.2 ± 11.5	5.00 – 54.0

SD, Standard deviation; IL-6, Interleukin-6.

**Figure 2:**
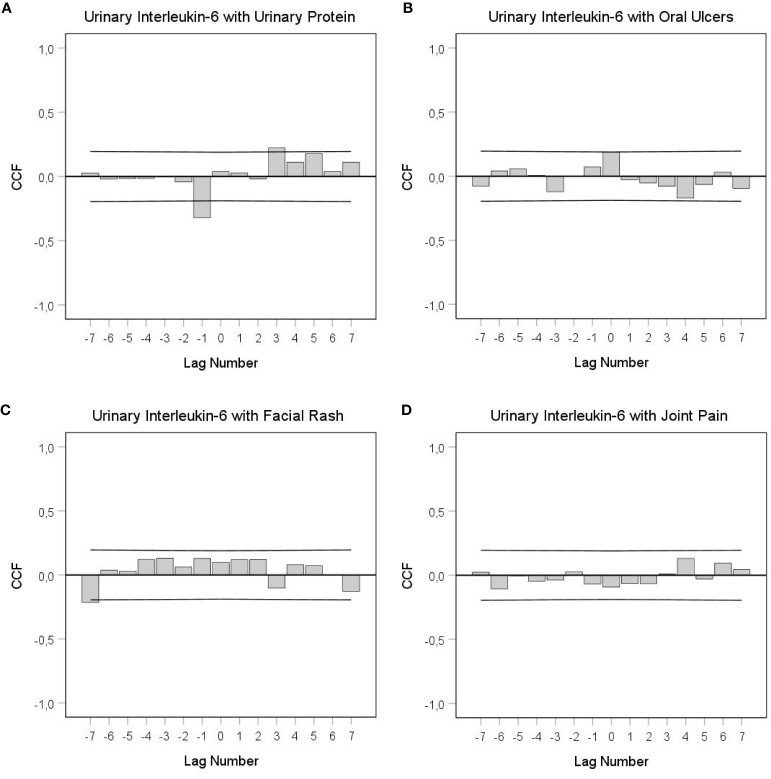
Cross-correlational functions (CCF) between urinary IL-6 levels and SLE-specific symptoms. **(A)** IL-6 with urinary protein, **(B)** IL-6 with oral ulcers, **(C)** IL-6 with facial rash, **(D)** IL-6 with small joint pain. Each lag represents a time interval of 12 h. Cross-correlation coefficients (bars) that reach the upper or lower limits of the 95% confidence intervals (lines) are significant at *p* < 0.05. *A* positive lag significance means that urinary IL-6 levels precede SLE-related symptoms; a negative lag significance means that SLE-related symptoms precede urinary IL-6 levels. Lag0 in this study can mean concurrency, positive lag (within 12 h) or negative lag (within 12 h). Clearly in **(A)**, but perhaps also in **(B)**, there is a change in the sign of the cross-correlation function between positive and negative lags, which indicates negative feedback loops.

**Table 2** shows a summary of the results of this evaluation. The urinary IL-6 time series is best described by an ARIMA model with stochastic as well as deterministic seasonal components corresponding to an 8-lag (96 h) rhythm. The cross-correlogram shown in **Figure 2A** reveals that increased urinary IL-6 concentrations significantly preceded increased urinary protein levels by 36–48 h (+lag3: *r*=+.225; *p*=.017) and that, in the opposite direction of effect, increased urinary protein levels significantly preceded decreased urinary IL-6 concentrations by 12–24 h (–lag1: *r*=–.322; *p*<.001). **Figure 2B** shows that increased urinary IL-6 levels co-occurred with increased oral ulceration (lag0: *r*=+.186; *p*=.049); after 48–60 h, however, IL-6 increases showed a strong tendency to precede decreases in oral ulceration (+lag4: r=–.170; *p*=.072). **Figure 2C** shows that increases in facial rash preceded decreases in urinary IL-6 concentrations by 84–96 h (–lag7: *r*=–.215; *p*=.023). As to fatigue, increased urinary IL-6 levels co-occurred with decreased fatigue (lag0: *r*=–.193; *p=*.042); after 84–96 h, however, IL-6 increases preceded increased fatigue (+lag7: *r*=–.189; *p*=.046) (data not shown). Finally, joint pain, another specific SLE symptom (**Figure 2D**), as well as tiredness and body temperature did not significantly correlate with urinary IL-6 levels, in either direction of effect.

**Table 2:** Summary of findings including ARIMA models and cross-correlation results between IL-6 concentrations and SLE-specific and SLE-nonspecific symptoms.

	Urinary IL-6 SAR(2), deterministic season, s=8, sqt		
**Urinary protein** (0,0,0), cube root	–lag1: r=–.322; *p*<.001		+lag3: r=+.225; *p*=.017
**Oral ulceration** not modeled		± lag0: r=.186; *p*=.049	+lag4: r=–.170; *n.s.*
**Facial rash** not modeled	–lag7: r=–.215; *p*=.023		
**Joint pain** AR(1), deterministic trend **Fatigue** deterministic trend **Tiredness** SMA(4), ln **Body temperature** deterministic season, s=2, ln		± lag0: r=–.193; *p*=.042	+lag7: r=.189; *p*=.046

+Lag means that IL-6 levels precede SLE symptom, –lag means that SLE symptom precedes IL-6 levels. Lag0 in this study can mean concurrency, positive lag (within 12 h) or negative lag (within 12 h).

IL-6, interleukin-6; AR, Autoregressive; SMA, Seasonal Moving Average; SAR, Seasonal Autoregressive; s, seasonality; n.s., not significant; sqt, square root; ln, natural logarithm.

## Discussion

While the involvement of IL-6 in B and T cell immunopathology of SLE is undisputed (26, 27), it still remains to be determined whether IL-6 is related to immune-associated ACR symptoms in SLE (4, 5). Accordingly, the special design of this study (e.g. time-series analysis on 112 12 h measurements) allowed us to show in a patient with SLE i) normal or even reduced mean urinary IL-6 concentrations (see **Table 1**) (17), ii) an 8-lag (96 h) circasemiseptan (about-half-weekly) (28) rhythmic pattern in the urinary IL-6 time series (see **Table 2**), and iii) clear interdependencies between the 12 h variations in urinary IL-6 concentrations and the 12 h variations in SLE symptoms. Specifically, cross-correlational analyses revealed that IL-6 either co-occurred with SLE symptoms (oral ulceration, fatigue), preceded (urinary protein) or followed (urinary protein, facial rash, fatigue) SLE symptoms, with temporal delays of up to 96 h. Such complex interrelations between IL-6 and SLE symptoms are new to autoimmune research and need careful interpretation. Specifically, findings from time series analyses cannot be compared easily with findings from conventional group statistics, which typically do not provide information on temporal delays, temporal patterns and directions of effect (15).

Given that proteinuria is one of the key features of lupus nephritis, our finding of an increase in urinary protein levels following IL-6 increases after 36–48 h confirms, in principle, results from laboratory studies. For example, in an experimental study on IL-6-knockout mice, Cash and colleagues (29) observed delayed lupus nephritis with a marked reduction of proteinuria compared to IL-6-intact control mice. The positive correlation between IL-6 levels and oral ulceration at lag 0 found in this study is also in line with group studies on this topic. Marques and colleagues, for example, showed a stronger positive expression of IL-6 in mucosal biopsies of lupus patients compared to the specimens of normal controls (30).

With regard to fatigue, our study found a negative correlation at lag 0 between urinary IL-6 levels and fatigue intensities as well as a positive correlation at +lag 7 (data not shown). This positive correlation at +lag 7, although describing a long temporal delay of 84–96 h, is principally in line with current experimental literature demonstrating that IL-6 triggers increases in fatigue and other symptoms of so-called sickness behavior (16). The other IL-6 result in regard to fatigue, namely the negative correlation at lag 0, is not in line with conventional research but is consistent with other findings from our working group. In studies on breast cancer patients, for example, we recently showed that increases in urinary IL-6 concentrations preceded fatigue decreases by 48–60 h (31) and that increased levels of urinary neopterin preceded fatigue increases by 24 h (32) and 60–72 h (33). Neopterin is a cellular immune parameter closely linked to the Th1 immunity (22). IL-6 has been shown to have well-defined anti-inflammatory properties and to promote Th2 responses often opposing Th1 activity (34). Thus, the current study’s finding of a decrease in fatigue co-occurring with IL-6 increases (in SLE) both replicates our previous findings on the temporal IL-6–fatigue relation (in breast cancer) (31) and is in line with our previous observations on the temporal relation between neopterin and fatigue (in breast cancer) (32, 33).

This study not only showed that urinary IL-6 changes co-occurred with or preceded changes in SLE symptoms but also that, in the opposite direction of effect, SLE symptoms preceded IL-6. Such findings are especially difficult to interpret from a conventional research perspective when, as is the case for urinary IL-6 and urinary protein, significances in both directions of effect (negative and positive lag) are found in the CCF (**Figure 2A**). We are convinced that such findings can only be interpreted properly when they are not considered in isolation. Instead, a broader look at the dynamic and possibly functional interdependencies between variables is required (18). In this regard, both long time delays and bidirectional effects between time series variables might indicate feedback mechanisms under real-life conditions (18, 35). Indeed, the cross-correlational constellation seen in **Figure 2A**, in which a negative (positive) value in one process becomes a positive (negative) value after having interacted with the other process, and vice versa, is an indicator of a negative feedback loop in the SLE patient under study (18, 35, 36). This negative feedback loop between urinary IL-6 and urinary protein could be read as follows: Increased protein clearance from circulation might have either per se or *via* an as yet unknown mechanism led to decreased IL-6 concentrations after 12–24 h (see the negative lag and negative correlation in **Figure 2A**), and suppressed IL-6 levels could have then resulted in decreased protein clearance with a temporal delay of 36–48 h (see the positive lag and positive correlation in **Figure 2A**).

The CCF between IL-6 and oral ulceration in **Figure 2B** shows a significantly positive correlation at lag 0 and a strong tendency toward negative significance at +lag 4. Similarly, the CCF between IL-6 and fatigue shows a significantly negative correlation at lag 0 and a positive significance at +lag 7 (data not shown). Both CCFs might indicate negative feedback loops when the following two conditions are met: i) In our study, a lag 0 significance can mean either that two variables are correlated without any directional effect between them or that one variable preceded the other within a time frame of 12 h. For a feedback loop, therefore, we need to assume that oral ulceration and/or fatigue preceded urinary IL-6 concentration changes by up to 12 h; ii) as to the relation between IL-6 and oral ulcers, the non-significant negative correlation at +lag 4 is in fact a meaningful finding when we keep in mind that both significant coefficients and temporal patterns of [non-significant] coefficients are important in the interpretation of integrative single-case studies (15).

Interestingly, the results on IL-6, urinary protein and oral ulcerations show inverted dynamics compared to a previous evaluation of the same integrative single-case study focusing on sTNF-R55 (18). In that evaluation, elevated urinary sTNF-R55 concentrations were preceded by increases in urinary protein and decreases in oral ulcers and were followed by decreases in urinary protein and increases in oral ulcers. In the current evaluation, by contrast, increased levels of urinary IL-6 were preceded by decreases in urinary protein and increases in oral ulcers and were followed by increases in urinary protein and decreases in oral ulcers. These findings indicate a counterregulatory temporal dynamic between sTNF-R55 and IL-6, which might be due to the mutual inhibition of Th1/Th2 subsets or to different pathways that these messenger molecules take (classical signaling/trans-signaling). In our two evaluations, therefore, we found cytokine markers of different Th subsets positively and negatively correlated with SLE symptoms at different points in time, thereby reinforcing the theory that SLE activity is a consequence of disturbed immunological balance (7, 9). Furthermore, these temporal dynamics might explain the inconsistencies in previous reports on the Th1/Th2 ratio in SLE (8–12).

Our current finding of a decrease in urinary IL-6 concentrations 84–96 h after increases in facial rash may be attributable to the emotionally painful experience of having a visible rash on the patient’s face (37). In this regard, the patient’s facial rash may have been a stressor that triggered a decrease in urinary IL-6 levels after 84–96 h (see **Figure 2C**). This reaction is similar to the evidence of stress-mediated neopterin responses found in this and other integrative single-case studies (15, 21). Specifically, in another evaluation of this patient (21), emotionally painful incidents were followed by ultimate increases in urinary neopterin concentrations after 60–72 h. As noted above, neopterin is a Th1 indicator (22); thus, the stress-mediated effects of neopterin oppose those of IL-6, again underscoring the different functional roles of neopterin and IL-6 with regard to the Th1/Th2 dichotomy.

Unlike findings from conventional group research (16), this integrative single-case study showed no significant effect of IL-6 on facial rash, small joint pain, tiredness and body temperature (see **Table 2**). Some of the null findings of this study could be attributed to the fact that no objective measurement of SLE symptoms was applied. This approach is based on the assumption that interfering too much in a patient’s everyday reality endangers the high ecological validity of this kind of investigation. For example, appointments with a specialist every 12 h to objectively measure the symptoms under investigation would have dominated the patient’s everyday routine. Furthermore, regular meetings with a specialist could have influenced the patient’s symptoms through placebo and/or nocebo effects. Such measures, therefore, would have interfered with the natural ebb and flow of the dynamic relation between immune factors and SLE symptoms. Nonetheless, in future studies, photographs of the skin taken by the patient in 12 h intervals would be a useful addition to objectify mucosal and skin lesions.

A further limitation of this evaluation is that only IL-6 was examined. Investigating additional inflammatory parameters (e.g. IL-1α, IL-1β) (16) could yield further insides, e.g. into the relation between immune factors and SLE symptoms as well as into Th1/Th2 regulation in SLE. The same holds true for transcription factors responsible for T-cell differentiation, which could be assessed in future studies through their measurement in urinary sediment.

For further evaluation of possible feedback processes between immune factors and SLE symptoms, multivariate time-series statistics (e.g. vector autoregressive modelling, impulse response analysis) could help to identify Granger causality and to properly disentangle the temporal sequence of the events within such regulatory circuits (38).

This study has exploratory character, and findings are based on only one patient (n=1). Therefore, we do not yet know whether the relations between immune factors and SLE symptoms found in this patient in disease remission also apply for patients experiencing acute disease activity. Thus, replications are needed before firm conclusions can be drawn.

Nevertheless, a considerable advantage of the integrative single-case study design is that it enables us to account for the dynamic nature of neuroimmunological processes (e.g. temporal delays and patterns, feedback mechanisms) under real-life conditions. These basic insights into the dysfunctional physiology of SLE would not be possible applying conventional laboratory and/or quasi-experimental approaches to this topic. Moreover, our results on the dynamic relation between cytokine levels and SLE symptoms call into question the use of pre-post designs in complex clinical research topics. The following considerations support this assertion: (1) A lack of conventional statistical correlation between a cytokine level and an SLE symptom (analogue to a lag 0 correlation) does not automatically mean that the cytokine is not connected with this symptom; (2) a single significant correlation between a cytokine level and an SLE symptom does not allow inferences on the functional role of a cytokine; (3) assuming that delayed effects between cytokine levels and SLE symptoms may differ across patients, an averaging of results would lead to inconsistencies and a lack of generalizability.

## Data Availability Statement

The raw data supporting the conclusions of this article will be made available by the authors, without undue reservation.

## Ethics Statement

The study, involving a human participant, was reviewed and approved by the Ethics Committee of the Medical Faculty of the University of Innsbruck. The patient provided her written informed consent to participate in this study. She also gave written informed consent for the publication of any potentially identifiable data included in this article.

## Author Contributions

CS: development of the study design, statistical analysis, interpretation of findings, writing of the manuscript. LS: interpretation of findings, writing of the manuscript. EC: interpretation of findings, writing of the manuscript. PK: patient recruitment, clinical and diagnostic work. NS: clinical and diagnostic work. FO-P: statistical analysis. MS-K: urinary protein measurement. DF: urinary IL-6 and creatinine measurement. All authors contributed to the article and approved the submitted version.

## Funding

Financial support for this research was provided by a grant from the National Bank of Austria (No. 6990).

## Conflict of Interest

The authors declare that the research was conducted in the absence of any commercial or financial relationships that could be construed as a potential conflict of interest.

## Publisher’s Note

All claims expressed in this article are solely those of the authors and do not necessarily represent those of their affiliated organizations, or those of the publisher, the editors and the reviewers. Any product that may be evaluated in this article, or claim that may be made by its manufacturer, is not guaranteed or endorsed by the publisher.
